# Benchmarking consensus model quality assessment for protein fold recognition

**DOI:** 10.1186/1471-2105-8-345

**Published:** 2007-09-18

**Authors:** Liam J McGuffin

**Affiliations:** 1 The School of Biological Sciences, University of Reading, Whiteknights, Reading RG6 6AS, UK

## Abstract

**Background:**

Selecting the highest quality 3D model of a protein structure from a number of alternatives remains an important challenge in the field of structural bioinformatics. Many Model Quality Assessment Programs (MQAPs) have been developed which adopt various strategies in order to tackle this problem, ranging from the so called "true" MQAPs capable of producing a single energy score based on a single model, to methods which rely on structural comparisons of multiple models or additional information from meta-servers. However, it is clear that no current method can separate the highest accuracy models from the lowest consistently. In this paper, a number of the top performing MQAP methods are benchmarked in the context of the potential value that they add to protein fold recognition. Two novel methods are also described: ModSSEA, which based on the alignment of predicted secondary structure elements and ModFOLD which combines several true MQAP methods using an artificial neural network.

**Results:**

The ModSSEA method is found to be an effective model quality assessment program for ranking multiple models from many servers, however further accuracy can be gained by using the consensus approach of ModFOLD. The ModFOLD method is shown to significantly outperform the true MQAPs tested and is competitive with methods which make use of clustering or additional information from multiple servers. Several of the true MQAPs are also shown to add value to most individual fold recognition servers by improving model selection, when applied as a post filter in order to re-rank models.

**Conclusion:**

MQAPs should be benchmarked appropriately for the practical context in which they are intended to be used. Clustering based methods are the top performing MQAPs where many models are available from many servers; however, they often do not add value to individual fold recognition servers when limited models are available. Conversely, the true MQAP methods tested can often be used as effective post filters for re-ranking few models from individual fold recognition servers and further improvements can be achieved using a consensus of these methods.

## Background

It is clear that one of the remaining challenges hindering the progress of protein fold recognition and comparative modelling is the selection of the highest quality 3D model of a protein structure from a number of alternatives [1]. The identification of appropriate templates used for building models has been significantly improved both through profile-profile alignments and meta-servers, to the extent that traditional threading methods are becoming less popular for fold recognition. Increasingly, for the majority of sequences with unknown structures, the problem is no longer one of template identification; rather it is the selection of the sequence to structure alignment that produces the most accurate model.

A number of methods have been developed over recent years in order to estimate the quality of models and improve selection. A popular technique has been to use methods such as PROCHECK [2] and WHATCHECK[3] in order to evaluate stereochemistry quality following comparative modelling. These methods were developed in order to check the extent to which a model deviates from real X-ray structures based on a number of observed measures. However, such evaluations are often insufficient to differentiate between stereochemically correct models. Traditionally, a variety of energy-based programs have been developed more specifically for the discrimination of native-like models from decoy structures. These programs were based either on empirically derived physical energy functions or statistical potentials derived from the analysis of known structures[4]. For some time, methods such as PROSAII [5] and VERIFY3D [6] have been in popular use for rating model quality. More recently, methods such as PROQ [7], FRST [8] and MODCHECK [9] have proved to be more effective at enhancing model selection.

During the 4^th ^Critical Assessment of Fully Automated Structure Prediction (CAFASP4), such methods were collectively termed as Model Quality Assessment Programs (MQAPs) and a number of them were evaluated in a blind assessment [10]. For the purposes of CAFASP4, an MQAP was defined as a program which took as its input a single model and which outputted a single score representing the quality of that model. Developers were encouraged to submit MQAPs as executables, which were subsequently used to evaluate models by the assessors.

More recently, quality assessment (QA) was incorporated as a new "manual" prediction category in the 7^th ^Critical Assessment of Techniques for Protein Structure Prediction (CASP7) [11]. The QA category was divided into two sub categories QMODE 1 referring to the prediction of the overall model quality and QMODE 2, in which the quality of individual residues in the model was predicted. In the QMODE 1 category, the format of the new experiment allowed users to run their methods in-house and then submit a list of server models with their associated predicted model quality scores. While this new format had certain advantages, it also allowed more flexibility in the type of methods which could be used for quality assessment. For example, this format allowed methods to be used which could not be evaluated as "true" MQAPs in the original sense, such as meta-servers approaches which may have used the clustering of multiple models or incorporated additional information about the confidence of models from the fold recognition servers.

In this paper, several of the top performing MQAPs are benchmarked in order to gauge their value in the enhancement of protein fold recognition. A number of top performing "true" MQAP methods are compared against some of the best clustering and meta-server approaches. In addition, two novel methods, which can be described as true MQAPs according to the original definition, are also benchmarked. Firstly, the ModSSEA method which is based on the secondary structure element alignment (SSEA) score previously benchmarked [12] and incorporated into versions of mGenTHREADER [13] and nFOLD [14]. Secondly, ModFOLD which combines the output scores from the ProQ methods[15], the MODCHECK method [9] and the ModSSEA method using an artificial neural network.

## Results and discussion

### Measurement of the correlation of predicted and observed model quality

**Figure 1:**
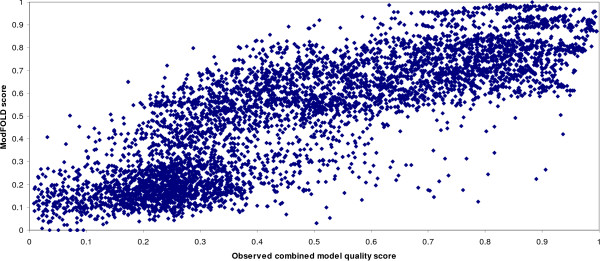
**Predicted model quality scores versus observed model quality scores**. The ModFOLD scores are plotted against the observed combined model quality scores ((TM-score+MaxSub+GTD)/3), for models submitted by the automated fold recognition servers to the CASP7 tertiary structure category (TS1 and AL1 models have been included).

The official CASP7 assessment of MQAP methods in the QMODE1 category involved measuring the performance of methods based on the correlation coefficients between predicted and observed model quality scores. In this section, the analysis is repeated both on a global and target-by-target basis. In Figure 1, each point on the plot represents a model submitted by a server to the CASP7 experiment. The models from all targets have been pooled together and so the "global correlation" is shown. The ModFOLD output score is clearly shown to correlate well with observed mean model quality score.

In Table 1, the global measures of Spearman's rank correlation coefficients (*ρ*) between predicted and observed model quality scores are shown for a number of the top performing MQAP methods. The Spearman's rank correlation is used in this analysis, as the data are not always found to be linear and normally distributed. The results shown here confirm the results in the official CASP7 assessment and show the LEE method and the ModFOLD method outperforming the other methods tested at CASP7 in terms of the global measure of correlation. Interestingly, the 3D-Jury method, which was not entered in the official assessment, is shown to outperform the LEE method based on all observed model quality scoring methods. The ModFOLD consensus approach appears to be working in this benchmark, as it is shown to outperform the individual constituent methods (MODCHECK, PROQMX, PROQLG and ModSSEA). The ModSSEA method, which was not individually benchmarked in the official assessment, also appears to be competitive with the established individual "true" MQAPs, which are capable of producing a single score based on a single model.

**Table 1:** Global measures of the Spearman's rank correlation coefficients (ρ)

	TM-score	MaxSub	GDT	Combined
3D-Jury^†^	0.955	0.924	0.925	0.943
LEE*^†^	0.943	0.903	0.909	0.926
ModFOLD	0.843	0.807	0.807	0.825
PROQ*	0.828	0.764	0.759	0.789
Pcons*^†^	0.803	0.773	0.765	0.786
ProQ-MX	0.779	0.755	0.751	0.768
ModSSEA	0.744	0.736	0.742	0.747
MODCHECK	0.729	0.659	0.658	0.686
ProQ-LG	0.688	0.651	0.640	0.665

All models are pooled together and the *ρ *is measured between predicted and observed model quality scores. The combined observed model quality score was also calculated for each individual model e.g. mean score for each model (TM-score+MaxSub+GTD)/3.*The MQAP scores for these methods were downloaded from CASP7 website; all other MQAP methods were run in house during the CASP7 experiment. †MQAP methods which rely on the comparison of multiple models or include additional information from multiple servers; all other methods are capable of producing a single score based on a single model.

The results in Table 2 again show the Spearman's rank correlation coefficients for each method, but in this instance the rho values are calculated for each target separately and then the mean overall rho value is taken. It is clear that the ordering of methods has changed and this was also shown to occur in the official assessment. The 3D-Jury method and the LEE method are still ranked as the top performing methods but there is a re-ordering of the other methods. Contrary to the results shown in Table 1, it would appear that there is no value from using the consensus approach of the ModFOLD method. How can these contradictory results be explained?

**Table 2:** Target-by-target measures of the Spearman's rank correlation coefficients (ρ)

	TM-score	MaxSub	GDT	Combined
3D-Jury^†^	0.870	0.818	0.857	0.862
LEE*^†^	0.793	0.734	0.771	0.779
Pcons*^†^	0.732	0.752	0.754	0.754
MODCHECK	0.574	0.568	0.587	0.584
PROQ*	0.557	0.575	0.580	0.576
ModFOLD	0.550	0.546	0.556	0.550
ModSSEA	0.506	0.501	0.520	0.516
ProQ-MX	0.412	0.444	0.444	0.438
ProQ-LG	0.289	0.340	0.326	0.320

Target-by-target measure – *ρ *is measured using the models for each target separately and the overall mean score is calculated. The combined observed model quality score was also calculated for each individual model e.g. mean score for each model (TM-score+MaxSub+GTD)/3. *The MQAP scores for these methods were downloaded from CASP7 website; all other MQAP methods were run in house during the CASP7 experiment. †MQAP methods which rely on the comparison of multiple models or include additional information from multiple servers; all other methods are capable of producing a single score based on a single model.

**Figure 2:**
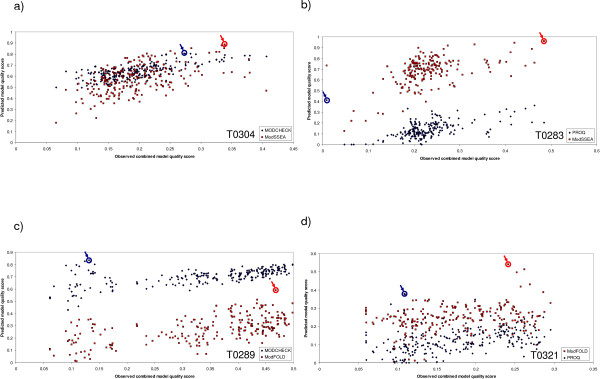
**Examples showing the difficulty with relying on correlation coefficients as performance measures**. Predicted model quality scores are plotted against the observed combined model quality scores on a target-by-target basis, for models submitted by the automated fold recognition servers to the CASP7 tertiary structure category (AL and TS models are included). a) The scaled MODCHECK scores are compared with the ModSSEA scores for the target T0304 models. The Spearman's rank correlation coefficient (ρ) between the MODCHECK scores and observed model quality scores is 0.66 and the observed model quality of the top ranked model (*m*) is 0.27 (the data point is circled in blue). The correlation coefficient for the ModSSEA method is lower (*ρ *= 0.50), however the quality of the top ranked model is higher (*m *= 0.34) (the data point is circled in red). b) The ProQ scores are compared with the ModSSEA scores for the target T0283 models. For ProQ *ρ *= 0.50 is and *m *= 0.01, whereas for ModSSEA, *ρ *= 0.40 is and *m *= 0.48. c) The scaled MODCHECK scores are compared with the ModFOLD scores for the target T0289 models. For MODCHECK, *ρ *= 0.61 is and *m *= 0.13, whereas for ModFOLD *ρ *= 0.53 is and *m *= 0.47. d) The ProQ scores are compared with the ModFOLD scores for the target T0321 models. For ProQ, *ρ *= 0.48 is and *m *= 0.11, whereas for ModFOLD, *ρ *= 0.17 is and *m *= 0.24.

The results in Figure 1 appear to show a roughly linear relationship between the predicted and observed model quality scores with few outliers based on the global measure where the models are pooled together for all targets. However, when the results are examined for individual targets (Figure 2) the relationship is often non-linear, the data are not always normally distributed and there are often a proportionately greater number of outliers which can influence the rho values. In developing MQAPs for the improvement of fold recognition the primary goal is to select the highest quality model as possible given a number of alternative models. Does the measurement of correlation coefficient on a target-by-target basis always help us to distinguish the best method for selecting the top model?

In Figure 2 (a-d), the scores from ModSSEA and ModFOLD are compared against MODCHECK and ProQ for four example CASP7 template based modelling targets. In these examples the rho values are higher for the MODCHECK and ProQ methods, however it can be seen that the observed quality scores for the top ranked models (which have been denoted *m *here) are shown to be higher for the ModFOLD and ModSSEA methods. Of course, there are also several cases where the the rho values for MODCHECK and ProQ are lower yet the *m *scores are higher than either ModFOLD or ModSSEA. Indeed by testing on a target-by-target basis, it was found that, on average, for each individual CASP7 target, the MQAP with the highest correlation coefficient between observed and predicted model quality was most often *not *the method with highest observed quality of the top ranked model.

From the scatter plots in Figure 2 it is apparent that the correlation between observed and predicted model quality may not necessarily be the best measure of performance if we are interested in methods which can identify the highest quality models. In real situations, developers and users of fold recognition servers would arguably be most concerned with the selection of the best model from a number of alternatives for a given target. The comparison of correlations coefficients should not necessarily replace the individual examination of the data. However, the individual examination of data for each method and for each individual target may not always be practical. It is therefore suggested that a more appropriate measure of the usefulness would be to simply measure the observed model quality of the top ranked models for each target (*m*) when benchmarking MQAPs for fold recognition.

### Measurement of the observed model quality of the top ranked models (*m*)

Table 3 shows the cumulative model quality scores that can be achieved if each MQAP method is used to rank the top models from all servers for each target (results are highlighted in bold). In other words, the *m *scores are taken from each MQAP for each target and then the scores are added together. Higher cumulative observed model quality scores (Σ*m*) can be achieved using the ModFOLD and ModSSEA methods than using the other true MQAPs, which are capable of producing a single score based on a single model (ProQ, ProQ-LG, PROQ-MX and MODCHECK).

**Table 3:** Cumulative observed model quality scores for each MQAP (TS1 and AL1 models)

	TM-score	MaxSub	GDT	Combined
**Maximum MQAP Score**	**62.30**	**52.98**	**56.25**	**57.18**
Zhang-Server_TS1	58.21	48.77	52.03	53.00
**3D-Jury**^†^	**58.02**	**48.32**	**51.96**	**52.77**
Pcons*^†^	**55.55**	**47.00**	**50.08**	**50.87**
LEE*^†^	**55.20**	**45.77**	**49.60**	**50.19**
**ModFOLD**	**55.39**	**45.47**	**49.62**	**50.16**
HHpred2_TS1	54.95	45.22	49.16	49.78
Pcons6_TS1	54.67	45.08	48.52	49.42
Pmodeller6_TS1	54.77	44.76	48.73	49.42
ROBETTA_TS1	54.92	44.43	48.85	49.40
CIRCLE_TS1	54.69	44.59	48.49	49.26
HHpred3_TS1	54.33	44.76	48.52	49.20
BayesHH_TS1	54.39	44.33	48.41	49.04
MetaTasser_TS1	55.17	43.80	48.15	49.04
HHpred1_TS1	54.18	44.48	48.04	48.90
UNI-EID_expm_TS1	54.06	44.58	47.95	48.86
**ModSSEA**	**54.30**	**43.88**	**48.35**	**48.84**
beautshot_TS1	54.37	44.25	47.75	48.79
FAMSD_TS1	54.07	44.08	48.05	48.73
PROQ*	**53.47**	**44.50**	**48.15**	**48.71**
RAPTOR-ACE_TS1	54.05	43.80	47.69	48.52
FAMS_TS1	53.84	43.70	47.84	48.46
SP3_TS1	53.51	43.48	47.41	48.13
SP4_TS1	53.44	43.19	47.11	47.91
shub_TS1	53.35	43.31	46.87	47.84
RAPTOR_TS1	53.48	42.88	47.16	47.84
UNI-EID_bnmx_TS1	52.33	43.72	46.88	47.64
beautshotbase_TS1	52.46	43.05	46.59	47.37
RAPTORESS_TS1	53.17	42.44	46.46	47.36
FUNCTION_TS1	52.75	42.59	46.57	47.30
SPARKS2_TS1	52.47	42.49	46.19	47.05
**PROQ-LG**	**51.49**	**43.04**	**46.43**	**46.99**
3Dpro_TS1	51.81	42.16	46.34	46.77
FOLDpro_TS1	51.77	42.06	46.10	46.64
GeneSilicoMetaServer_TS1	51.75	42.09	45.87	46.57
UNI-EID_sfst_AL1.pdb	50.39	42.55	45.37	46.10
PROTINFO_TS1	51.28	41.36	45.60	46.08
Ma-OPUS-server_TS1	51.23	40.96	45.30	45.83
SAM_T06_server_TS1	51.35	40.66	45.12	45.71
**PROQ-MX**	**49.89**	**41.60**	**44.89**	**45.46**
PROTINFO-AB_TS1	50.64	40.65	44.65	45.32
Phyre-2_TS1	50.26	40.32	44.38	44.99
ROKKY_TS1	49.66	40.42	44.16	44.75
mGen-3D_TS1	49.29	40.15	44.22	44.55
Bilab-ENABLE_TS1	49.59	39.16	43.26	44.00
SAM-T02_AL1.pdb	48.13	40.12	43.03	43.76
LOOPP_TS1	48.44	38.64	42.73	43.27
FUGUE_AL1.pdb	47.55	38.79	42.53	42.96
nFOLD_TS1	47.40	38.46	41.95	42.60
keasar-server_TS1	47.84	38.20	41.59	42.54
Phyre-1_TS1	46.87	38.16	41.63	42.22
**MODCHECK**	**47.03**	**37.76**	**41.65**	**42.15**
NN_PUT_lab_TS1	46.95	37.72	41.26	41.98
CaspIta-FOX_TS1	46.53	37.47	41.01	41.67
FUGMOD_TS1	46.37	37.42	41.10	41.63
FORTE1_AL1.pdb	46.51	37.06	40.66	41.41
FORTE2_AL1.pdb	46.30	36.89	40.56	41.25
3D-JIGSAW_POPULUS_TS1	44.74	35.44	39.34	39.84
karypis.srv_TS1	44.43	35.20	38.95	39.53
3D-JIGSAW_RECOM_TS1	43.70	35.55	38.84	39.36
3D-JIGSAW_TS1	43.53	34.50	38.37	38.80
SAM-T99_AL1.pdb	42.60	35.81	37.64	38.69
karypis.srv.2_TS1	42.77	33.54	37.50	37.94
Huber-Torda-Server_TS1	41.78	34.40	37.21	37.80
forecast-s_AL1.pdb	41.00	33.38	36.48	36.95
Distill_TS1	39.75	27.26	31.94	32.98
Ma-OPUS-server2_TS1	33.35	26.75	29.77	29.96
panther2_TS1	28.87	23.67	25.85	26.13
CPHmodels_TS1	27.75	23.49	24.55	25.26
Frankenstein_TS1	23.55	17.66	20.33	20.52
gtg_AL1.pdb	20.55	16.66	17.81	18.34
ABIpro_TS1	21.88	12.35	17.45	17.22
MIG_FROST_AL1.pdb	16.68	12.11	14.75	14.51
FPSOLVER-SERVER_TS1	14.91	6.78	10.97	10.89
karypis.srv.4_TS1	14.71	6.55	10.66	10.64
POMYSL_TS1	9.64	6.00	8.35	8.00
panther3_TS1	5.75	4.58	5.05	5.12
MIG_FROST_FLEX_AL1.pdb	1.05	0.97	1.07	1.03

Results in **bold **indicate the cumulative observed model quality scores of the top ranked models for each target (Σ*m*) obtained by using each MQAP method to rank the *top *models from all fold recognition servers. The maximum achievable MQAP score – obtained by consistently selecting the best model for each target – is also highlighted. All other results are based on the cumulative scores of the TS1 or AL1 models from each fold recognition server taking part in the automated category at CASP7. Each column indicates the method for measuring the observed model quality. Scores are sorted by the combined observed model quality. *The MQAP scores for these methods were downloaded from CASP7 website; all other MQAP methods were run in house during the CASP7 experiment. †MQAP methods which rely on the comparison of multiple models or additional information from multiple servers; all other methods are capable of producing a single score based on a single model.

The methods which rely on the comparison of multiple models and/or additional information from multiple servers (3D-Jury, LEE and Pcons) are shown to greatly outperform the individual true MQAPs, however the consensus approach taken by ModFOLD is shown to be competitive.

The cumulative model quality scores of the TS1 or AL1 models from each fold recognition server are also shown in Table 3. The 3D-Jury, Pcons, LEE and ModFOLD methods achieve a higher cumulative score than all fold recognition servers except the Zhang-Server. It must be noted that the cumulative scores which can be achieved by ranking models using any of the existing MQAP methods are still far lower than the maximum achievable MQAP score obtained if the best model were to be consistently selected for each target. Table 4 shows the cumulative observed model quality scores if MQAP methods are used to rank all models from all servers. For all of the methods, except the 3D-Jury method, there is a reduction in the cumulative observed model quality. The LEE method outperforms the Pcons method but the relative performance of all other methods is unchanged. However, are the differences in *m *scores from the different MQAP methods significant?

**Table 4:** Cumulative observed model quality scores for each MQAP (all models)

	TM-score	MaxSub	GDT	Combined
3D-Jury^†^	58.22	48.19	52.21	52.87
LEE*^†^	55.17	45.66	49.59	50.14
Pcons*^†^	54.47	45.81	49.20	49.82
ModFOLD	54.26	44.36	48.57	49.06
ModSSEA	53.73	43.24	47.65	48.21
PROQ*	51.20	42.82	45.99	46.67
PROQLG	49.32	41.62	44.63	45.19
PROQMX	46.93	39.04	42.23	42.73
MODCHECK	43.76	34.85	38.66	39.09

The cumulative observed model quality scores of the top ranked models for each target (Σ*m*) obtained by using each MQAP method to rank *all *models from all fold recognition servers.*The MQAP scores for these methods were downloaded from CASP7 website; all other MQAP methods were run in house during the CASP7 experiment. †MQAP methods which rely on the comparison of multiple models or additional information from multiple servers; all other methods are capable of producing a single score based on a single model.

Often the differences observed between methods in terms of cumulative observed model quality scores (Σ*m*), may not be significant. The results in Tables 5, 6, 7 are provided to demonstrate that the rankings between methods shown in Table 3 and 4 are only relevant if a significant difference is observed according to the Wilcoxon signed rank sum tests. The p-values for Wilcoxon signed ranks sum tests comparing the MQAP methods are shown in Tables 5, 6, 7. The null hypothesis is that the observed model quality scores of the top ranked models (*m*) from *method x *are less than or equal to those of *method y*. The alternative hypothesis is that the *m *scores for *method x *are greater than those of *method y*.

**Table 5:** Calculated p-values for Wilcoxon signed rank sum tests (TM-score)

	*Method x*								
	
*Method y*	MODCHECK	ProQ-MX	ProQ-LG	ProQ*	ModSSEA	ModFOLD	Pcons*^†^	LEE*^†^	3D-Jury†
MODCHECK		0.33	0.10	9.21E-03	9.25E-04	6.54E-05	2.85E-05	3.80E-08	1.89E-12
ProQ-MX	0.67		4.04E-02	3.34E-03	1.49E-06	1.14E-07	1.83E-07	3.49E-09	1.42E-12
ProQ-LG	0.91	0.96		2.35E-02	5.82E-05	1.54E-05	2.51E-07	5.88E-09	4.31E-13
ProQ*	0.99	1.00	0.98		4.29E-02	1.15E-02	3.43E-05	2.67E-06	8.17E-11
ModSSEA	1.00	1.00	1.00	0.96		0.26	2.53E-02	5.08E-03	1.32E-07
ModFOLD	1.00	1.00	1.00	0.99	0.75		0.05	2.76E-02	3.15E-07
Pcons*^†^	1.00	1.00	1.00	1.00	0.98	0.95		0.38	1.07E-04
LEE*^†^	1.00	1.00	1.00	1.00	1.00	0.97	0.63		5.02E-05
3D-Jury^†^	1.00	1.00	1.00	1.00	1.00	1.00	1.00	1.00	

The different MQAP methods are compared in terms of the observed model quality of the top ranked models for each target. H_0 _= *m*_*x *_≤ *m*_*y*_, H_1 _= *m*_*x *_> *m*_*y*_, where H_0 _is the null hypothesis; H_1 _is the alternative hypothesis; *m*_*x *_is the observed model quality of models selected by *Method x *and *m*_*y *_is the observed model quality of models selected by *Method y *according to the TM-score. * The MQAP scores for these methods were downloaded from CASP7 website; all other MQAP methods were run in house during the CASP7 experiment. † MQAP methods which rely on the comparison of multiple models or additional information from multiple servers; all other methods are capable of producing a single score based on a single model.

**Table 6:** Calculated p-values for Wilcoxon signed rank sum tests (MaxSub)

	*Method x*								
	
*Method y*	MODCHECK	ProQ-MX	ProQ-LG	ProQ*^†^	ModSSEA	ModFOLD	Pcons*^†^	LEE*^†^	3D-Jury^†^
MODCHECK		0.05	9.47E-03	2.99E-03	1.56E-03	2.61E-05	1.92E-06	4.09E-08	4.02E-11
ProQ-MX	0.95		2.74E-02	1.36E-02	2.70E-03	1.54E-05	1.80E-06	7.16E-07	5.21E-11
ProQ-LG	0.99	0.97		0.18	0.12	1.10E-02	9.48E-06	8.15E-06	2.95E-11
ProQ*	1.00	0.99	0.82		0.28	0.06	7.67E-05	3.74E-05	3.67E-08
ModSSEA	1.00	1.00	0.88	0.72		0.08	7.84E-04	1.01E-03	5.11E-07
ModFOLD	1.00	1.00	0.99	0.94	0.93		1.41E-02	3.80E-02	1.45E-05
Pcons*^†^	1.00	1.00	1.00	1.00	1.00	0.99		0.57	5.30E-03
LEE*^†^	1.00	1.00	1.00	1.00	1.00	0.96	0.43		2.28E-03
3D-Jury^†^	1.00	1.00	1.00	1.00	1.00	1.00	0.99	1.00	

The different MQAP methods are compared in terms of the observed model quality of the top ranked models for each target. H_0 _= *m*_*x *_≤ *m*_*y*_, H_1 _= *m*_*x *_> *m*_*y*_, where H_0 _is the null hypothesis; H_1 _is the alternative hypothesis; *m*_*x *_is the observed model quality of models selected by *Method x *and *m*_*y *_is the observed model quality of models selected by *Method y *according to the MaxSub score. * The MQAP scores for these methods were downloaded from CASP7 website; all other MQAP methods were run in house during the CASP7 experiment. † MQAP methods which rely on the comparison of multiple models or additional information from multiple servers; all other methods are capable of producing a single score based on a single model.

**Table 7:** Calculated p-values for Wilcoxon signed rank sum tests (GDT)

	*Method x*								
	
*Method y*	MODCHECK	ProQ-MX	ProQ-LG	ProQ*	ModSSEA	ModFOLD	Pcons*^†^	LEE*^†^	3D-Jury^†^
MODCHECK		0.14	3.59E-02	5.99E-03	1.09E-03	9.83E-06	3.88E-06	4.69E-08	1.37E-11
ProQ-MX	0.87		4.77E-02	7.52E-03	3.85E-05	3.58E-07	7.98E-07	3.24E-08	3.38E-12
ProQ-LG	0.96	0.95		0.07	3.48E-03	1.05E-04	2.99E-07	6.41E-08	2.06E-13
ProQ*	0.99	0.99	0.93		0.14	1.03E-02	3.40E-05	8.83E-06	2.91E-09
ModSSEA	1.00	1.00	1.00	0.86		0.06	5.80E-03	2.53E-03	7.13E-08
ModFOLD	1.00	1.00	1.00	0.99	0.94		4.64E-02	0.06	2.90E-06
Pcons*^†^	1.00	1.00	1.00	1.00	0.99	0.95		0.43	1.47E-03
LEE*^†^	1.00	1.00	1.00	1.00	1.00	0.94	0.57		1.01E-03
3D-Jury^†^	1.00	1.00	1.00	1.00	1.00	1.00	1.00	1.00	

The different MQAP methods are compared in terms of the observed model quality of the top ranked models for each target. H_0 _= *m*_*x *_≤ *m*_*y*_, H_1 _= *m*_*x *_> *m*_*y*_, where H_0 _is the null hypothesis; H_1 _is the alternative hypothesis; *m*_*x *_is the observed model quality of models selected by *Method x *and *m*_*y *_is the observed model quality of models selected by *Method y *according to the GDT score. * The MQAP scores for these methods were downloaded from CASP7 website; all other MQAP methods were run in house during the CASP7 experiment. † MQAP methods which rely on the comparison of multiple models or additional information from multiple servers; all other methods are capable of producing a single score based on a single model.

The top models selected using the 3D-Jury method are shown to be of significantly higher quality (p < 0.01) than those selected using any other method according to the TM-score, MaxSub score and GDT score. The top models selected using the ModFOLD method are of significantly higher quality than those of PROQ-MX, PROQ-LG and MODCHECK according to the TM-score (p < 0.01), MaxSub score (p < 0.05) and GDT score (p < 0.01) (Table 5, 6 and 7). According to the MaxSub score the top models selected by both LEE and Pcons are significantly higher quality (p < 0.05) than those selected by ModFOLD (Table 6).

However, there is no significant increase in the quality of the top models selected by Pcons over those selected by ModFOLD according to the TM-score (Table 5). In addition there is no significant increase in the quality of models selected by the LEE method over the ModFOLD method according to GDT score (Table 7). Variation in the predicted secondary structures or other input parameters would explain the observed differences between the in house version of ProQ-LG and the ProQ scores downloaded from the CASP7 website, however the overall difference between scores is not shown to be significant (Table 5, 6 and 7).

The ModSSEA method was developed independently for the CASP7 experiment, prior to the publication of the comparable method developed by Eramian *et al*. [16]. Although the two methods are similar in that they both compare the DSSP assigned secondary structure of the model against the PSIPRED predicted secondary structure of the target, they differ in their scoring. The two methods were found to show differences in cumulative observed model quality scores (a mean difference of 1.08), however none of these were found to be significant according to the Wilcoxon signed rank sum test with each measure of observed model quality: using the TM-score the p-value was 0.1765, using the MaxSub score the p-value was 0.1625 and using the GDT score the p-value was 0.1355.

### Measurement of the confidence in the true MQAP output scores

One of the advantages of the so called "true" MQAPs (e.g. ProQ, MODCHECK, ModSSEA and ModFOLD) over clustering methods (e.g. 3D-Jury and LEE) and those which use also use information from multiple fold recognition servers (e.g. Pcons), is that they provide a single consistent and absolute score for each individual model. This means that the models from different protein targets can be directly compared with one another on the same predicted model quality scale. Conversely, with clustering methods the scores for a given model are potentially variable as they are dependent on the relationship between many models of the same target protein. Similarly, the information which can be obtained from multiple fold recognition servers may vary from target to target. Therefore, the predicted model quality scores between different targets may not be directly comparable as they do not directly relate to model quality.

**Figure 3:**
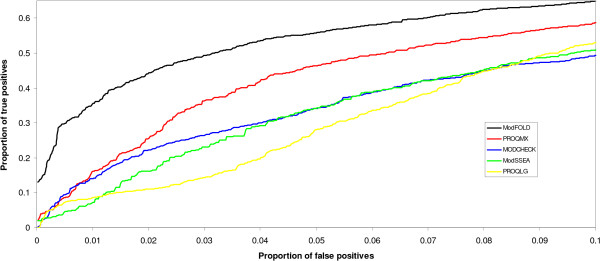
**A benchmark of the consistency of the ModFOLD predicted model quality score**. The proportion of true positives is plotted against the proportion of false positives. The CASP7 fold recognition server models (21714 models from 87 targets -see methods) were ranked by decreasing predicted model quality score using ModFOLD and the different MQAP methods that make up the ModFOLD method. False positives were defined as models with TM-scores ≤ 0.5, indicating models that have a different fold to the native structure. True positives were defined as models with TM-scores > 0.5 indicating models that share the same fold as the native structure [17]. The plot shows the proportion of true positives at the region of < = 10% false positives.

The consistency of the output scores from the true MQAPs is useful in the context of the structural annotation of proteomes, where it is important to be able estimate the coverage of modelled proteins at a particular level of confidence. In order to be able to measure the confidence of a prediction we must be able to directly compare model quality scores from different protein targets. In Figure 3, the confidence in output scores from the 5 true MQAPs are compared by ranking all models according to predicted model quality and then plotting the number of true positives versus false positives, according to observed model quality, as the output scores decrease. A TM-score of 0.5 is used as a stringent cut-off to define false positives. Models above this cut-off are likely to share the same fold as the native structure [17]. A higher true positive rate is shown for the ModFOLD method than for the other MQAP methods tested at low rates of false positives. This indicates that we can have a higher confidence in the ModFOLD output score over the other true MQAP methods, implying that ModFOLD method should be a more useful method in the context of proteome annotation using fold recognition. In other words, a higher coverage of high quality models can be selected with a lower number of errors.

### Benchmarking on standard decoy sets

It could be argued that data sets such as the CASP7 server models provide a more appropriate and larger test set for the benchmarking MQAP methods, particularly in the practical context of fold recognition. Methods such as ModFOLD, are often developed and tested for the selection of the best real fold recognition model rather than for the detection of the native fold amongst a set of artificial decoys.

However, in order to enable direct comparisons with additional published methods, benchmarking was carried out the using three commonly used standard decoy sets from the Decoys 'R' Us [18] database (4state_reduced [19], lattice_ssfit [20] and LMDS [21]) and the results are shown in Table 8. The ModFOLD method appears to be competitive with other MQAPs using the standard decoy sets according to standard measures of performance such as the rank and Z-score of the native structure (see Tosatto's recent paper for a comparison of methods using these sets and scoring [8]). However, due to the smaller number of targets in these sets it is not often possible to calculate significant differences between the methods. It is also observed that the relative performance of methods appears to be dependent on which dataset is used, although it is not possible to draw sound conclusions from this data.

**Table 8:** Benchmarking based on three standard decoy sets from the Decoys 'R' Us database

	*4state_reduced*		*Lattice_ssfit*		*LMDS*	
*Method*	*Z-score*	*Rank 1*	*Z-score*	*Rank 1*	*Z-score*	*Rank 1*

ModFOLD	3.74	5/7	10.12	7/8	3.33	5/10
PROQ-LG	3.73	3/7	11.19	7/8	1.91	1/10
PROQ-MX	3.44	4/7	18.15	7/8	2.15	3/10
MODCHECK	2.20	3/7	5.05	8/8	1.64	3/10
ModSSEA	1.95	3/7	4.31	6/8	1.62	3/10

*Rank 1 *– the number of native structures correctly ranked first by each method out of the total proteins in decoy set; *Z-score *– the average Z-scores calculated as the distance in standard deviations from the MQAP score of the native structure to the mean score of the decoy set.

### Measurement of the added value of re-ranking few models from individual servers

It is clear from the cumulative observed model quality scores (Σ*m*) in Tables 3 and 4 and Wilcoxon signed rank sum tests (Tables 5, 6 and 7) that if we have many models from multiple servers then the best MQAP methods to use are those which carry out comparisons between multiple models for the same target (e.g. 3D-Jury). However, what if only few models are available from an individual server? Can developers and users of individual fold recognition servers gain any added value from re-ranking their models using an MQAP method?

**Figure 4:**
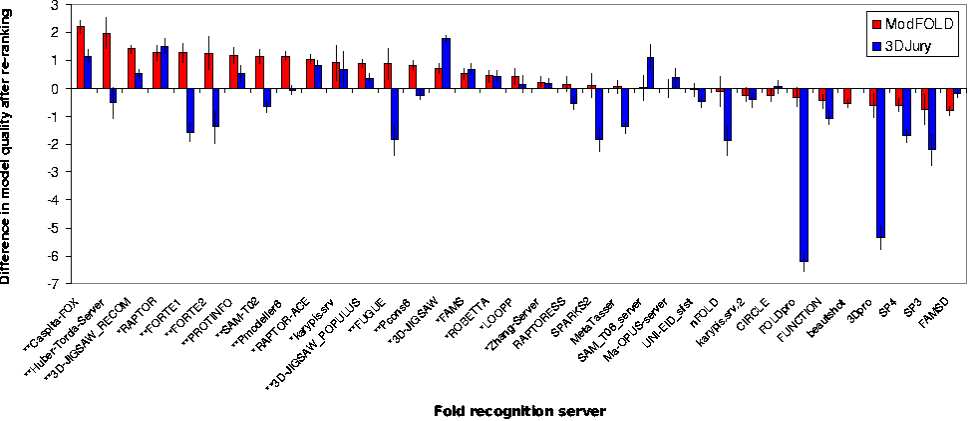
**The added value of re-ranking models**. The difference in the cumulative observed model quality score of the top ranked models is shown after the 5 models for each target provided by each server are re-ranked using the ModFOLD or 3D-Jury methods. Each bar represents Σ(*m*_*i*_*-m*_*j*_*)*, where *m*_*i *_is the observed model quality of the top ranked model after the 5 server models are re-ranked and *m*_*j *_is the observed model quality of the original top ranked model submitted by the server. N.B. Only the common subset of servers which had submitted 5 models for all targets are included in the plot. The error bars show the standard error of the mean observed quality. Overall there is a mean *increase *of 0.44 in the cumulative observed model quality of the top ranked models if the ModFOLD method is used to re-rank the models provided by individual servers, however, there is a mean *decrease *of 0.56 if models are re-ranked using the 3D-Jury method (see Table 9). On the x axis, the first asterisk indicates a fold recognition server where the quality of the top ranking model can be significantly improved. An additional asterisk indicates a significant improvement of the ModFOLD method over the 3D-Jury method.

Figure 4 shows the difference in observed mean model quality score, or the "added value", obtained if the ModFOLD method is used to select the best model out of the 5 submitted by each individual server compared against using the 3D-Jury clustering approach. For most of the fold recognition servers tested, the model quality scores can be improved if ModFOLD is used as a post filter in order to re-rank models. However, on average the model quality score is decreased if a clustering approach, such as 3D-Jury, is used to re-rank models from the individual servers.

**Table 9:** The added value of re-ranking models measured by cumulative observed model quality

	TM-score	MaxSub	GDT	Combined
ModFOLD	0.42	0.42	0.47	0.44
ProQLG	0.27	0.32	0.33	0.31
PROQ*	0.23	0.34	0.30	0.29
MODCHECK	0.25	0.32	0.30	0.29
ProQMX	-0.09	0.05	0.00	-0.01
3D-Jury^†^	-0.49	-0.61	-0.59	-0.56
ModSSEA	-1.12	-1.06	-0.97	-1.05
Random	-3.61	-3.56	-3.48	-3.55

The mean difference in cumulative observed model quality scores if each MQAP method is used to re-rank the models from each individual fold recognition server. The results achieved from a random re-ranking of models from each server (random assignment of scores between 0 and 1) are also shown for comparison. * The official predicted MQAP scores for these methods were downloaded from CASP7 website; all other MQAP methods were run in house during the CASP7 experiment. † MQAP methods which rely on the comparison of multiple models or additional information from multiple servers; all other methods are capable of producing a single score for a single model.

In the case of the CaspIta-FOX server, the cumulative quality score of the top selected models can be improved from 41.67 to 43.88, using ModFOLD, which would improve the overall ranking of the method by 8 places in Table 3. The Zhang-Server score can also be marginally improved upon from 53.00 to 53.23 if ModFOLD is used to re-rank models. Several individual servers can also be improved using the 3D-Jury method; however, for the majority of servers, there is less benefit to be gained from re-ranking very few models using the clustering approach.

On average the cumulative observed model quality score of an individual server is improved by 0.44 if the ModFOLD method is used to re-rank the 5 submitted models (Table 9). Table 9 also shows that on average the quality score of the top selected model is improved for individual servers using the ProQ, ProQ-LG and MODCHECK methods, confirming our previous results [9]. The ProQ-MX, ModSSEA and 3D-Jury methods on average show an overall decrease in the quality of the top selected models from each server, if these methods are used as post filters to re-rank models.

Table 10 shows the proportion of servers which can be improved by using each MQAP method to re-rank submitted models, according to each observed model quality score. The ModFOLD method is shown to improve ~66% (23/35) of the servers tested according to all measures of observed model quality and the ProQ method improves ~69% (24/35), according to the combined observed model quality score.

**Table 10:** The added value of re-ranking measured by the proportion of improved servers

	TM-score	MaxSub	GDT	Combined
PROQ*	0.69	0.71	0.69	0.69
ModFOLD	0.66	0.66	0.66	0.66
ProQLG	0.60	0.60	0.60	0.63
MODCHECK	0.46	0.51	0.57	0.60
ProQMX	0.43	0.46	0.43	0.49
3D-Jury^†^	0.44	0.38	0.47	0.44
ModSSEA	0.20	0.17	0.23	0.20
Random	0.03	0.03	0.06	0.03

The proportion of the fold recognition servers (out of the 35 tested) which have been improved according to observed model quality scores through the re-ranking of models using each MQAP method. The results achieved from a random re-ranking of models from each server (random assignment of scores between 0 and 1) are also shown for comparison. * The official predicted MQAP scores for these methods were downloaded from CASP7 website; all other MQAP methods were run in house during the CASP7 experiment. † MQAP methods which rely on the comparison of multiple models or additional information from multiple servers; all other methods are capable of producing a single score for a single model.

What if we were also to use the information from the original server ranking in addition to the MQAP scores? Can further improvements to model ranking be made by using this information as an additional weighting to the MQAP score? The results in Table 11 and Table 12 show the additional improvement to model rankings made by combining the information from the original server ranking with that of the MQAP score. In this benchmark, models initially ranked by a server as the top model achieve a higher additional score than models initially ranked last. A useful additional score was found to be (6-r)/40, where r is the initial server ranking of the model between 1 and 5 (e.g. the additional score for a TS1 model would be 0.125, a TS2 model would have an additional score of 0.1 etc.).

**Table 11:** The added value of re-ranking with weighted scores (cumulative observed model quality)

	TM-score	MaxSub	GDT	Combined
ModFOLD	0.69	0.67	0.70	0.69
ProQLG	0.56	0.56	0.57	0.56
PROQ*	0.55	0.57	0.55	0.56
MODCHECK	0.48	0.47	0.46	0.47
ProQMX	0.19	0.33	0.33	0.28
3D-Jury^†^	0.01	-0.04	-0.04	-0.02
ModSSEA	-0.07	-0.02	0.00	-0.03
Random	-3.41	-3.62	-3.78	-3.58

Similar to Table 9, however the original server ranking is also considered and added to the score as an extra weighting ((6-r)/40, where r is the original server ranking between 1 and 5). The results achieved from a random re-ranking of models from each server (random assignment of scores between 0 and 1) are also shown for comparison. * The official predicted MQAP scores for these methods were downloaded from CASP7 website; all other MQAP methods were run in house during the CASP7 experiment. † MQAP methods which rely on the comparison of multiple models or additional information from multiple servers; all other methods are capable of producing a single score for a single model.

**Table 12:** The added value of re-ranking with weighted scores (proportion of improved servers)

	TM-score	MaxSub	GDT	Combined
MODCHECK	0.74	0.80	0.77	0.77
ModFOLD	0.74	0.74	0.71	0.74
ProQLG	0.71	0.69	0.71	0.71
PROQ*	0.71	0.74	0.69	0.69
ProQMX	0.57	0.63	0.63	0.63
ModSSEA	0.51	0.51	0.51	0.51
3D-Jury^†^	0.54	0.49	0.57	0.49
Random	0.03	0.03	0.06	0.06

Similar to Table 10, however the original server ranking is also considered and added to the score as an extra weighting ((6-r)/40, where r is the original server ranking between 1 and 5). The results achieved from a random re-ranking of models from each server (random assignment of scores between 0 and 1) are also shown for comparison. * The official predicted MQAP scores for these methods were downloaded from CASP7 website; all other MQAP methods were run in house during the CASP7 experiment. † MQAP methods which rely on the comparison of multiple models or additional information from multiple servers; all other methods are capable of producing a single score for a single model.

Table 11 shows that on average the cumulative observed model quality score for an individual server can be increased by 0.69, if the initial ranking score is added to the ModFOLD score and used as a post filter to re-rank models. The number of servers improved using the combined score also increases to 74% (26/35) (Table 12). For all other MQAP methods the scores are also be improved by using information from the server in addition to the MQAP scoring. This is a similar technique to that used in the Pcons method, albeit used here with a more basic scoring scheme and benchmarked on the few models produced by individual servers, rather than many models from multiple servers.

This is a stringent benchmark as there are few models to choose from each individual server. This means that there is less information to be gained from a comparison of the structural features shared between models. Therefore, the clustering approach (3D-Jury) does not perform well at this task. The ModSSEA method also performs badly at this task as it is also dependent on differentiating models based on structural features. If there is conservation of secondary structure among the top few models from the same server, then the ModSSEA method will perform badly. Indeed, many servers already include secondary structure scores and so the top models provided by the same server are often likely to share similar secondary structures. The value of randomly selecting the top models (through the assignment of a random score between 0 and 1) has also been included in Tables 9 to 12. A random selection of the top model on average shows a marked decrease in model quality as the probability of a correctly selecting the top model for a given target is 0.2.

## Conclusion

The consensus MQAP method (ModFOLD) is shown to be competitive with methods which use clustering of multiple models or information from multiple servers (LEE and Pcons) according to the cumulative observed model quality scores of the top ranked models (Σ*m*). Furthermore, according to this benchmark the ModFOLD method significantly outperforms some of the best "true" MQAP methods tested here (ProQ-MX, ProQ-LG and MODCHECK), all of which produce single consistent scores based on a single model.

Benchmarking based on correlation coefficients is not always helpful in measuring the usefulness of MQAP methods. There is not always a linear relationship between the MQAP score and the observed model quality score and scores for an individual target may not be normally distributed. Even with the non-parametric test, outliers can affect the results and so the correlation coefficient should not replace the individual examination of the data. It is therefore proposed that simply measuring the observed model quality scores of the top ranked model (*m*) on a target by target basis, or the cumulative scores (Σ*m*) over all targets, may be more useful for benchmarking MQAPs in the context of protein fold recognition, followed by measures of the statistical significance. In practical terms, predictors require the best model to be selected for a given target and so *m *is an appropriate measure of the performance of an MQAP method in this context.

If there are many models available from multiple fold recognition servers then clustering models using the 3D-Jury approach is demonstrably the most effective tested method for ranking models. However, the method can perform poorly when there are very few models available and often no value is added by re-ranking of models from an individual sever. Furthermore, methods such as 3D-Jury, LEE and Pcons may not produce consistent scores and therefore scores of models from different targets cannot be directly compared against one another. Clustering methods, such as 3D-Jury, are also computationally intensive and the CPU time required for calculating a score increases quadratically with number of available models.

The so called "true" MQAP methods tested here (ModFOLD, ModSSEA, MODCHECK and the ProQ methods) are less computationally intensive as they consider only the individual model when producing a score. Therefore, the computational time for these methods scales linearly with the number of available models. They are also demonstrated here to add value to predictions when used as a post filter to re-rank even very few models from individual fold recognition servers.

In the context of a CASP assessment it is clear that the MQAP methods that make use of clustering of multiple models are currently superior to true MQAP methods that score individual models. Server developers wishing to perform well in CASP will therefore be more likely to use and develop the former methods as they will have access to many models produced by many different servers. However, in a practical context, experimentalists may have collected only very few models from the limited number of publicly accessible servers which remain available outside the context of CASP. Therefore, experimentalists would be advised to consider using the true MQAP methods in order to rank their models prior to investing valuable time in the laboratory. However, it is clear that there is room for the further improvement of both the true MQAP methods and the methods which make use of clustering and multiple servers, in the selection of the highest quality models. This is evidenced by the maximum possible score that could be achieved by consistently selecting the highest quality model.

## Methods

A number of the top performing Model Quality Assessment Programs (MQAPs) were benchmarked using the fold recognition models submitted by servers in the CASP7 experiment. Several of the "true" MQAP methods, which can produce a single score based on a single model alone (MODCHECK and three versions of ProQ), were benchmarked against those methods which make use of the clustering of multiple models or information from multiple servers in order to calculate scores (3D-Jury, LEE and Pcons). In addition, two new true MQAP approaches were tested: ModSSEA, based on secondary structure element alignments and ModFOLD, a consensus of MODCHECK, ModSSEA and the ProQ methods.

### ProQ and MODCHECK

The ProQ [7] and MODCHECK [9] methods have been shown previously to be the amongst the most effective of the "true" MQAP methods according to benchmarking carried out in a previous study [9]. Executables for each program were downloaded [22] and run in-house individually on the test data (see below), using the default parameters. The ProQ method produced two output scores per model, ProQ-MX and ProQ-LG, which were benchmarked separately. The ProQ scores from the version submitted for the CASP7 model quality assessment (QMODE 1) category were also downloaded via CASP7 results website[23].

### ModSSEA

The ModSSEA method was developed as a novel model quality assessment program based on secondary structure element alignments (SSEA). The ModSSEA score was determined in essentially the same way as the SSEA score which have been previously benchmarked [12-14], however, the PSIPRED [24] predicted secondary structure of the target protein was aligned against the DSSP [25] assigned secondary structure of the model, as opposed to the secondary structure of a fold template. The ModSSEA score was incorporated along with the MODCHECK and ProQ scores into the ModFOLD method described below.

### ModFOLD

Predictions for the CASP7 model quality assessment (QMODE 1) category were generated using the ModFOLD method. The method was loosely based on the nFOLD protocol [14] and combined the output from a number of model quality assessment programs (MQAPs) using an artificial neural network. The scaled output scores from the in house versions of MODCHECK [9], ProQ-LG, ProQ-MX [7] and ModSSEA were used as inputs to a feed forward back propagation network. The neural network was then trained to discriminate between models based on the TM-score [26]. The neural network architecture used for ModFOLD simply consisted of four input neurons, four hidden neurons and a single output neuron. The models for the training set were built from mGenTHREADER [27] alignments to > 6200 fold templates using an in-house program, which simply mapped aligned residues in the target to the full backbone coordinates of the template and carried out renumbering. The target-template pairs were then generated from an all against all comparison of the sequences from non-redundant fold library. Sequences within the training set had BLAST [28] E-values > 0.01 and < 30% identity to one another.

The four selected MQAPs were used to predict the quality of each of the structural models in the training set. The resulting MQAP scores were scaled to the range 0–1 and were fed in to the input layer. The network was trained using the observed quality of each model, which was calculated using the TM-score. The resulting neural network weight matrix was saved and subsequently used to provide in-house consensus predictions of model quality.

### Pcons and LEE

The Pcons and LEE groups were the overall top performing groups at CASP7 according to the official assessment. The Pcons method has been described previously [15] and is widely used as a consensus fold recognition server. From the CASP7 abstracts it is understood that the method used by the LEE group was based on a combination of the clustering of models, an artificial neural network and energy functions. As the methods produced by these groups could not be tested in house, the scores submitted by these groups for the CASP7 model quality assessment (QMODE 1) category were downloaded via CASP7 results website [23].

### 3D-Jury

The 3D-Jury method [29] is a popular and effective method of clustering models which was not tested in the CASP7 model quality assessment category. However, the simplicity of the approach allows it to be run in-house easily for comparison against the leading methods. Therefore, for each target, the models were also scored using an in-house approach similar to that of the 3D-Jury method [29], however, TM-scores were used to determine the similarities between models rather than MaxSub scores (using the TM-score instead of the MaxSub score was found to give a marginally better performance).

### Testing Data

The fold recognition server models for each CASP7 target were downloaded via the CASP7 website [30]. The individual MQAPs which make up ModFOLD, were used to evaluate every server model (both AL and TS) for each CASP7 target. The ModFOLD predictions were then submitted to assessors prior to the expiry date for each target and therefore prior to the release of each experimental structure. After the CASP experiment, 87 of the non-cancelled official targets that had published experimental structures released into the PDB (as of 26/11/06) were used to provide a common set of models in order to benchmark the performance of each method.

In addition, several standard test sets were downloaded from the Decoys 'R' Us [18] database (4state_reduced [19], lattice_ssfit [20] and LMDS [21]) so that ModFOLD and ModSSEA may be compared with additional published methods. The ability of methods to identify the native structure from each set of decoys was tested using standard measures.

### Measuring observed model quality

The TM-score program [26] was used to generate the TM-scores, MaxSub scores [31] and GDT scores [32], which were used to measure the observed model quality for each individual model. The combined score was also calculated for each individual model i.e. the TM-score, MaxSub and GDT scores were calculated for each model and the mean score was then taken for each model separately.

### The ModFOLD server

The ModFOLD predictions were carried out entirely automatically for all targets throughout the CASP7 experiment. A web server has been implemented for the ModFOLD method, which is freely available for academic use [33]. The server accepts gzipped tar files of models – similar to the official CASP7 tarballs – and returns predictions in the CASP QA (QMODE1) format via email.

## Authors' contributions

LJM carried out the entire study.
